# Origins and implications of neglect of G6PD deficiency and primaquine toxicity in *Plasmodium vivax* malaria

**DOI:** 10.1179/2047773215Y.0000000016

**Published:** 2015-05

**Authors:** Kevin Baird

**Affiliations:** University of Oxford, UK and Indonesia

**Keywords:** *Plasmodium vivax*, G6PD deficiency, Primaquine, Anti-relapse therapy, History, Neglect

## Abstract

Most of the tens of millions of clinical attacks caused by *Plasmodium vivax* each year likely originate from dormant liver forms called hypnozoites. We do not systematically attack that reservoir because the only drug available, primaquine, is poorly suited to doing so. Primaquine was licenced for anti-relapse therapy in 1952 and became available despite threatening patients having an inborn deficiency of glucose-6-phosphate dehydrogenase (G6PD) with acute haemolytic anaemia. The standard method for screening G6PD deficiency, the fluorescent spot test, has proved impractical where most malaria patients live. The blind administration of daily primaquine is dangerous, but so too are the relapses invited by withholding treatment. Absent G6PD screening, providers must choose between risking harm by the parasite or its treatment. How did this dilemma escape redress in science, clinical medicine and public health? This review offers critical historic reflection on the neglect of this serious problem in the chemotherapy of *P. vivax.*

## Introduction

‘Doctors commonly get mixed up between absence of evidence and evidence of absence’. *Nassim Nicholas Taleb*

How humanity perceives a problem ultimately defines the nature and urgency of the solutions collectively applied to solving it. Drug-resistant *Plasmodium falciparum*, for example, commands priority and diligence in order to prevent loss of life due to failed therapy of that dangerous infection.^1^ The community of malariology undertakes vigorous efforts at understanding and systematically monitoring that problem in order to spare patients from poor outcomes.^2^,^3^ Failing to monitor antimalarial drug efficacy – and instead using drugs we hope still work, but may not – would be reckless and irresponsible. The life of the patient too often depends upon delivering therapies that cure falciparum malaria.^4^ This evidence-informed perception and appropriately vigorous response has not been applied for patients infected by *Plasmodium vivax*.

 Monitoring efficacy of chloroquine against the acute attack of vivax malaria is rarely undertaken.^5^ Endemic resistance in many areas was demonstrated over two decades ago,^6^ and is now highly prevalent across large swathes of Southeast Asia.^7^ Flawed reasoning largely explains the lack of vigour in monitoring drug resistance in *P. vivax*: although poor therapies may result in illness, infection by this benign species rarely causes loss of life. The false perception of harmlessness in this species eased the burden of responsibility to always deliver therapies that work. The conspicuous differences in management of the respective drug resistance problems in these two dominant *Plasmodium* species reflect our perceptions of the differing consequences of failed therapy between them.

In the case of *P. vivax* a second therapy, primaquine, is required to kill dormant liver stages called hypnozoites. Those stages, absent in *P. falciparum*, later awaken and provoke repeated clinical attacks of vivax malaria.^8^ There is no systematic surveillance undertaken to monitor the efficacy of this drug, despite more than 60 years of continuous use. The failure to gather evidence of sustained good efficacy of this drug, like chloroquine, also stems from the perception of relatively minor consequences with poor efficacy. The provision of presumptive primaquine therapy against such attacks in non-immune travellers, for example, has been viewed as optional.^9^–^12^ Providers seem content to risk post-travel attacks rather than offer primaquine therapy.^13^ Similarly, treating against relapse in patients living in endemic areas was long viewed as unnecessary – the 1981 treatment guidelines for malaria from the World Health Organisation (WHO) expressed, ‘*It is doubtful if radical treatment of vivax malaria is necessary if the patient lives in an endemic area where transmission of the infection continues and reinfection is likely*’.^14^

When weighed against the threat posed by daily primaquine in patients of unknown glucose-6-phosphate dehydrogenase (G6PD) status, the risk of later clinical attacks by a non-threatening species seemed a reasoned weighing of risk and benefit. Primaquine causes a mild to severe acute haemolytic anaemia in patients having an inborn deficiency of G6PD. This highly diverse X-linked disorder affects over 400 million people and occurs at an average prevalence of 8% in malaria endemic nations.^15^ Safety concerns demand screening out patients deficient in G6PD prior to offering daily primaquine therapy, especially where clinical monitoring is impractical. However, the only methods available for doing so require laboratory skills, special equipment, a cold chain for reagents and come at relatively high cost.^16^ The inability to distinguish G6PD-deficient from normal patients often results in all of them being denied access to primaquine therapy. The hypnozoite reservoir goes largely unchallenged, streaming new clinical attacks and onwards transmission into the human communities where it resides unmolested by primaquine.^17^,^18^

The absence of evidence that these practices actually caused injury or failed to mitigate harm dominates among other factors likely explaining the long persistence of poor access to safe therapy against relapse. Testing the hypothesis of harmlessness in *P. vivax*, demonstrating health dividends in withholding primaquine therapy, or proof of safety in offering it without G6PD screening – all would have offered materialised evidence of maximal benefit with minimal harm, but such evidence was not developed and followed. Instead, we carried on in these practices for over 60 years, apparently confident that the long absence of contrary evidence somehow affirmed the assumptions embedded within them.

But evidence of harm being done has emerged. That evidence, reviewed here, provides a wholly new perspective on vivax malaria and its management, and a corrective course is being vigorously advocated within the field of contemporary malariology. Understanding how this very significant problem stood for so long without attention from the communities of medicine, science and public health offers important historic insights informing future strategy on this and other human problems.

## Pernicious and Not Benign

**Figure 1.**
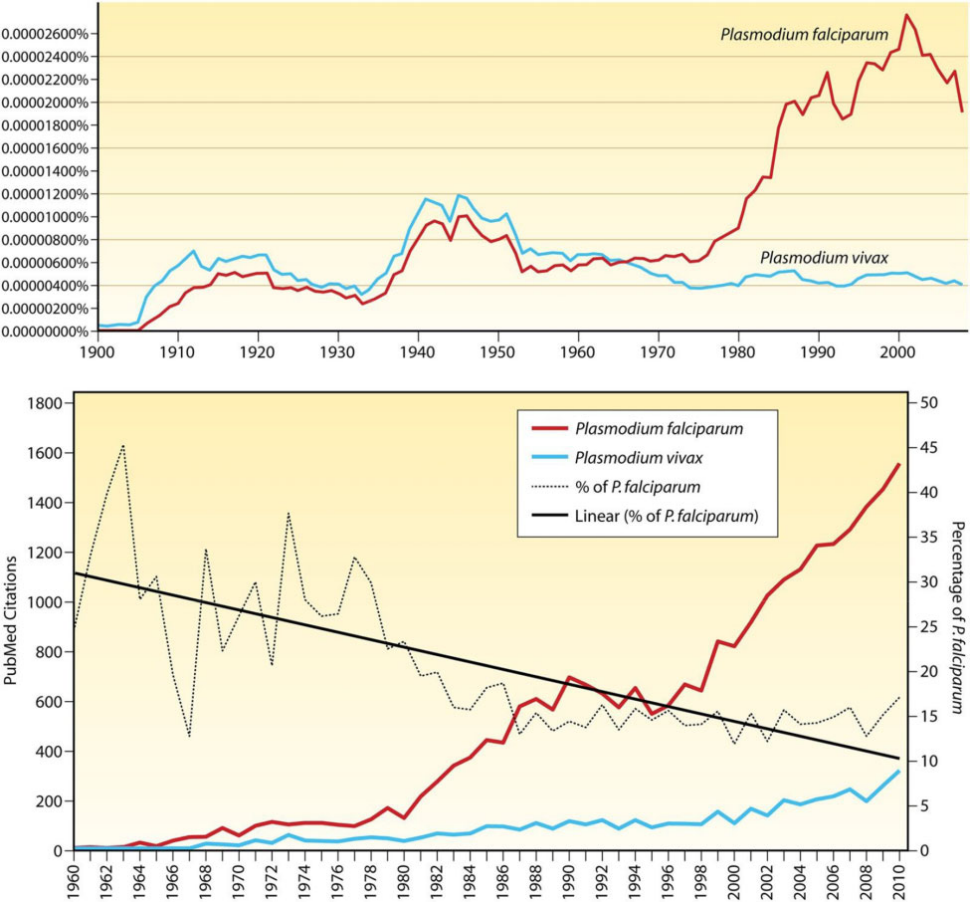
Graph illustrating the percentage of citations in books for *Plasmodium falciparum* and *Plasmodium vivax* from 1900 to 2008 by use of the tool at http://books.google.com/ngrams (1A, top), and citation data from PubMed since 1960 (1B, bottom) taken from Ref. 86 published under Creative Commons license. These graphs reproduced from Ref. 28 with the permission of Clinical Microbiology Reviews.

Discussion of the neglect of *P. vivax* and its management as a clinical and public health problem requires consideration of the intrinsic harmless character erroneously assigned to this species.^19^,^20^ The false dichotomy of benign versus malignant malarias, represented by *P. vivax* and *P. falciparum*, profoundly influenced contemporary malariology. Humanity naturally focussed resources and energies on the ‘harmful’ species as a greater priority than the ‘harmless’ species – a focus engendered by *P. falciparum* also being less complex and amenable to laboratory cultivation. *P. vivax* seemed to offer relatively minor scientific and health dividends as returns on the extraordinary difficulty of both basic research and successful treatment. *Plasmodium falciparum* has overwhelmingly dominated research agendas and outputs, to include both basic research papers and translational products of research (Fig. 1).

Recent evidence informs a view of *P. vivax* as pernicious rather than benign. An attack by *P. vivax* causes agonising daily paroxysms often accompanied by intractable nausea and vomiting, headache, myalgia and profound asthenia.^21^ Most patients are incapacitated for several days, even with prompt diagnosis and effective treatment. Chronic or repeated attacks by this parasite very often lead to severe anaemia and risk of death.^22^,^23^ Just a few weeks of untreated infection in non-immune adults drives haemoglobin levels below 5 g/dl,^24^ and young children in endemic zones are especially susceptible to threatening severe anaemia.^25^ Severe illness with acute infection includes lung injury with respiratory distress, kidney injury with renal dysfunction, hepatic dysfunction and jaundice, seizures/delirium/coma, severe thrombocytopaenia or circulatory collapse.^26^,^27^ The risk of patients in the hospitals of endemic settings being classified as severely ill with a primary diagnosis of vivax malaria approximates that in patients with falciparum malaria in the same hospitals, about 10%.^28^,^29^ In hospitals anywhere, the risk of death as an outcome with severe disease and a primary diagnosis of malaria is statistically indistinguishable between the two species, typically ranging between 5 and 15%.^28^–^30^ Those severe morbidity and mortality rates perhaps exclude from the denominators studies carried out where local *P. vivax* may be relatively non-threatening. Variable virulence linked to strain identity or access to good healthcare is known in this species.^29^ Nevertheless, no studies have affirmed the broad and deeply entrenched notion of *P. vivax* as intrinsically benign or harmless, and poor access to limited healthcare services is the rule where endemic vivax malaria occurs.

The above synopsis of the clinical consequences of infection by *P. vivax* only recently emerged. Contrast that current assessment with S.F. Kitchen's of 1949, ‘*this parasite* [*P. vivax*] *does not appear to possess, in the sense thatP. falciparum does, any attributes that induce perniciousness. It is therefore difficult to understand how it can, in the absence contributory factors, cause dangerous clinical states*’.^24^ Acknowledging that error lays the foundation for understanding the specific problem of the neglect of primaquine and G6PD deficiency as a serious obstacle to effective therapy.

## Benign Neglect

Alphonse Laveran described the protozoan aetiology of malaria in 1880, and by 1895 the three principal species of human plasmodia were described taxonomically. *P. falciparum, P. vivax* and *Plasmodium malariae* became objects of study in establishing taxonomic order in clinics applying a pre-Laveran taxonomy based on clinical manifestations.^24^ The clinical taxonomy recognised and exploited clear distinctions in the course and consequences of what malariologists of that era understood to be distinct malarial diseases.^31^ Some of those classes of malaria were very rarely fatal, while others were often lethal. The notion of benign and malignant malarias long predates knowledge of species identities.

Today, we recognise that all species of plasmodia infecting humans usually cause a relatively mild and non-threatening illness or even none at all (with naturally acquired immunity). Nonetheless, each of the five known species infecting humans – now including *Plasmodium ovale* and *Plasmodium knowlesi,*^32^,^33^ the latter being a zoonosis and very often threatening – may also turn deadly. But this understanding is recent, and for most of the 125 years since the plasmodia infecting us became known, we accepted species identity as ordaining malignant versus benign courses and consequences. A diagnosis of *P. falciparum* inspired fear and dread, whereas *P. vivax, P. malariae* or *P. ovale* evoked little concern regarding possible loss of life. This dichotomy profoundly shaped not only the science of malariology, but also broader views of malaria as a global health problem. The global malaria mortality burden came to be viewed as being caused almost entirely by a single species of parasite (*P. falciparum*) on a single continent (Africa) – and humanity focussed its ingenuity, energies and resources on those.^29^

What many considered the definitive text of malariology, Boyd's two volume set published in 1949, included chapters on the clinical course and consequence of the four human malaria species^24^ (excluding *P. knowlesi,* recognised as such only a decade ago^34^). The contributing malariologists all had lived through the transition from malaria endemic to non-endemic North America and Europe, and then many of them managed the Global Malaria Eradication Campaign of the 1950s and 1960s. Although a global nadir in endemic malaria occurred in the mid-1960s (exempting most of sub-Saharan Africa), the WHO abandoned the campaign in 1969 as untenable.^35^ Malariology in most developed nations contracted or vanished with local endemic transmission, and few protégés were cultivated. Many of the experienced malariologists of that era retired in the belief that malaria would soon be eradicated. We may thus appreciate Boyd's text and others like it as key links to what had been nearly lost wisdom in malariology.

The global resurgence of malaria that began during the 1970s eventually mobilised a new generation of malariologists in the 1980s and 1990s who possessed a great deal less direct experience. They turned to the knowledge recorded in texts like Boyd's. The title of that text certainly invited viewing it as authoritative: *Malariology: a Comprehensive Survey of All Aspects of This Group of Diseases from a Global Standpoint,* as did its extraordinary richness of technical detail across the promised broad array of the field.

In the chapter on the clinical course of *P. vivax* in humans, Kitchen described the species as intrinsically benign, biologically incapable of doing serious harm.^24^ He did so almost exclusively on the basis of the consistency of relatively very low-grade infection of peripheral blood; compared to *P. falciparum*; typically < 10 000/μl versus >100 000/μl in his experimentally infected study subjects being thus treated for neurosyphilis. Kitchen understood the fastidious and obligatory invasion of reticulocytes by merozoites of *P. vivax*, versus the promiscuous invasion of any red blood cell by those of *P. falciparum*. This created the impression of a self-limiting and benign infection versus a limitless replication in malignant fashion that marked infection by *P. falciparum* in his subjects. The dogma of benign versus malignant tertian malarias from the pre-Laveran classifications had been seemingly verified by Kitchen. However, no consideration had been given to the possibility of the bulk of harmful biomass of *P. vivax* occurring exterior to the vascular sinuses and being undetectable by examination of peripheral blood smears.^29^ No direct evidence informed that hypothesis of an inherently harmless species, and Kitchen rejected or set aside the evidence suggesting otherwise. Harmlessness in vivax malaria thus became a tenet of modern malariology.

Research on vivax malaria became sharply limited after the 1950s. It would not begin to rebound, as did research on *P. falciparum* beginning in the 1970s, until after the new millennium. During all this time, up to the present day, primaquine stood as the only therapy against relapse. The inability to provide it safely without G6PD screening failed to register as a problem in need of solving. Understanding that failure requires examining the genesis of primaquine therapy.

## Discovery of Anti-Relapse Therapy and G6PD Deficiency

The false perception of inherent harmlessness in *P. vivax* does not fully account for the inadequacy of primaquine against relapse. The sections to follow retrace the discovery and development of anti-relapse therapy and the linked discovery of G6PD deficiency. This history more directly informs the quest to understand the genesis of the exceedingly poor effectiveness of primaquine and its acceptance as a satisfactory therapeutic solution for *P. vivax* for over 60 years.

### Emergence of anti-relapse therapies

Excepting the herbal remedy for fevers later found to contain artemisinin, until the early twentieth century quinine represented the only therapeutic agent against acute malaria. Although in the late nineteenth century a synthetic aniline dye (methylene blue) cured acute malaria (in just two research subjects), no serious attempt at discovery of synthetic antimalarials was undertaken before about 1910. In that era, the Dutch consolidated their global monopoly on quinine production on Java in the East Indies (Indonesia). German scientists at the I.G. Farben Laboratories challenged that monopoly with a suite of synthetic antimalarial drugs. They began their search with methylene blue, systematically substituting various moieties to that core molecule and observing the effects on therapeutic activity (against *Plasmodium reticulum* in a Javanese finch model). This revolutionary work birthed medicinal chemistry and the methodology for discovery of chemotherapeutic agents.^36^,^37^

The first synthetic drug they brought to market (in 1927) was an 8-aminoquinoline they called plasmochin, later also known as pamaquine. They marketed it as therapy for the acute attack, but the drug soon earned a reputation as being dangerous, especially in non-Caucasians.^38^–^40^ There was no understanding of G6PD deficiency and 8-aminoquinoline haemolytic toxicity in that era, nor was there certain knowledge of latent liver stages of the plasmodia. When practitioners augmented lower doses of pamaquine with quinine, they aimed only at mitigating the toxicity of pamaquine for therapy of the acute attack. Patients thus treated for acute vivax malaria, it was noticed, were much less likely to suffer delayed attacks (relapses).^41^ Radical cure of vivax malaria had thus been unwittingly invented. Understanding of the specific activity of pamaquine against dormant liver stages came much later. None of the synthetic antimalarials seriously challenged the primacy of quinine until events denied access to it.

### Synthetic antimalarials and the Pacific War

**Figure 2.**
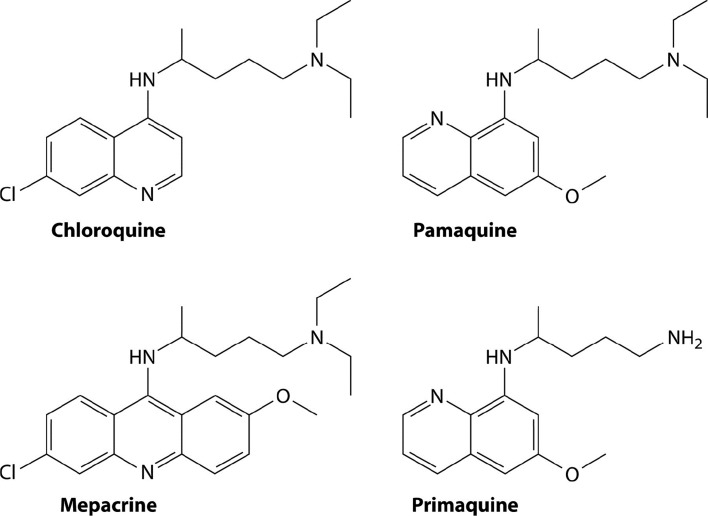
Chemical structures of the synthetic antimalarials created by I.G. Farben/Bayer in Germany during the 1920s and 1930s (pamaquine, mepacrine and chloroquine) and primaquine from the US Army during the 1940s. Reproduced from Ref. 6 with the permission of Clinical Microbiology Reviews.

In March 1942, the Imperial Japanese seized the Netherlands East Indies, including Java, where 95% of the global supply of quinine originated. The Allies rallied to manufacture a synthetic blood schizontocide called atabrine (or mepacrine, or quinacrine) and pamaquine, suspending the I.G. Farben/Bayer patent rights on those compounds. Figure 2 illustrates the chemical relatedness of these compounds, along with those of chloroquine and primaquine. The Allies faced a very serious malaria threat in the Pacific and the loss of access to quinine impelled them to aggressive corrective action. Threat of war spurred creation of the Board for Coordination of Malaria Studies in 1941 at the National Science Foundation in Washington, DC. That board mobilised enormous financial, scientific and clinical capital into antimalarial drug discovery and development.^42^

In the early 1940s, malariologists understood the phenomenon of relapse in *P. vivax* and the likely involvement of a dormant tissue stage (liver and hypnozoite as yet unknown as that tissue and stage), along with the apparent activity of pamaquine against those hypothesised latent tissue stages. The Allied war planners understood the threat relapse posed, not only to their troops in the field, but they also worried about several million returning troops reintroducing endemic malaria transmission to a North American continent on the verge of eliminating it. Attacking the dormant tissue stage was a very high priority, and they attempted doing so with an untried combination of drugs for radical cure: atabrine (blood schizontocide) and pamaquine (tissue schizontocide). An unexpected drug–drug interaction exacerbated the already marginal safety of pamaquine,^43^ and in 1943 the US Surgeon General ordered pamaquine not be used with atabrine against relapse.^44^ The ability to treat against relapse was lost at the moment of greatest need.

### Discovery of primaquine

The Board for the Coordination of Malaria Studies resolved to address this threatening problem and mobilised a search for a less toxic option to pamaquine.^45^ They ordered that only 8-aminoquinolines be considered in that search in order to assure speedy delivery of an agent certain to prevent relapse. In their aim of mitigating the toxicity of pamaquine, they had no understanding of the central role of G6PD deficiency in that problem. Their preclinical screening of 8-aminoquinoline candidates consisted of classical toxicity studies in rats and dogs, none being relevant to the core toxicity problem of innate susceptibility to acute haemolytic anaemia in some patients. About 2-dozen candidate compounds were thus selected and went to clinical trials in prisoner volunteers. The effort outlasted the war, and in 1950 primaquine emerged as having a slightly higher therapeutic index than pamaquine and other 8-aminoquinoline candidates.^46^

The original problem of pamaquine interaction with atabrine had been rendered irrelevant by the ‘discovery’ of chloroquine licenced for the treatment of acute malaria in 1946 – that was another I.G. Farben compound (they called it resochin) lifted by the Allies. Coatney detailed the extraordinary intrigue-filled tale of its development.^47^ In 1952, primaquine was licenced in the USA for radical cure of vivax malaria in combination with chloroquine for terminating the acute attack. The developers knew of the phenomenon of ‘primaquine sensitivity’ in their prisoner volunteers. Working with pamaquine and quinine during the war, they found that the effective total dose could be distributed over 14 days with good efficacy against relapse and mitigating the observed haemolysis among the pamaquine-sensitive subjects,^48^ predominantly African Americans.

In this history one may grasp that the chemical search leading to primaquine excluded the universe of chemical possibilities beyond the 8-aminoquinolines, and it could not select against the key toxicity problem with that class of compounds. No model of latent hypnozoites guided the process of winnowing candidates for superior therapeutic activity prior to trials in humans. This war-spurred and sharply limited drug discovery endeavour delivered a flawed and deficient product (one scarcely different from pamaquine in structure, Fig. 2), and one that remains the only therapy against relapse 63 years later. Misconstruing the seemingly non-threatening treatment largely explains the view of primaquine as a satisfactory solution for radical cure of vivax malaria, then and in the decades that followed.

### Discovery of G6PD deficiency

Most of the clinical trials leading to discovery of primaquine were conducted at the Stateville Penitentiary in Illinois using inmate volunteers.^49^ Investigators noted haemolytic sensitivity to 8-aminoquinolines (all of them evaluated) among African-American but not Caucasian prisoner volunteers.^50^ Meticulous clinical and laboratory investigation led to the identification of deficiency in erythrocytic G6PD as the basis of primaquine sensitivity in 1956.^51^ In that year, primaquine had already been in clinical use for over 5 years, principally among US troops in combat on the Korean Peninsula. The US military developers of primaquine had adapted to administering the therapy without knowledge of G6PD deficiency. They had already experienced the 15 mg daily dose of primaquine for 14 days, so effective against Korean *P. vivax* strains, as being sufficiently well tolerated by primaquine sensitive soldiers. Screening for G6PD deficiency thus seemed pointless and was not routinely done by the US Army (until about 2006).

During the 1960s, primaquine therapy against relapse was routinely administered without knowing G6PD status of the patient. Providers were warned to be alert to the risk of haemolytic anaemia and to stop dosing with onset of signs. This was the US Army dosing strategy developed before G6PD deficiency was known, and it found global application in WHO recommendations for radical cure of vivax malaria. As late as 1981 the WHO recommendation read, ‘*Reports on large numbers of patients treated with this regimen, even where G6PD deficiency is quite common, indicate that this regimen* [15 mg daily for 14 days] *is generally well tolerated and that hemolysis, when it occurs, is mild and self-limiting*’.^14^ This recommendation effectively refers to safety demonstrated by the absence of evidence of harm rather than materialised evidence of safety. The American experience with primaquine in a handful of otherwise healthy G6PD-deficient African-American men characterised as provoking a ‘mild and self-limiting’ haemolysis seemed universally applicable. The safety of primaquine without G6PD screening in broader human populations was presumed rather than demonstrated.

The great diversity of G6PD deficiency was only dimly understood in that era. Papers from the mid- to late-1960s began reporting G6PD variants of extreme sensitivity to primaquine relative to the African A −  variant. Severe reactions requiring transfusion, and some ending in death, appeared as case reports in the literature.^52^–^56^ Experimental primaquine challenge of subjects having the Mediterranean variant of G6PD deficiency affirmed relatively extreme sensitivity to primaquine compared to A − .^57^–^59^

**Figure 3.**
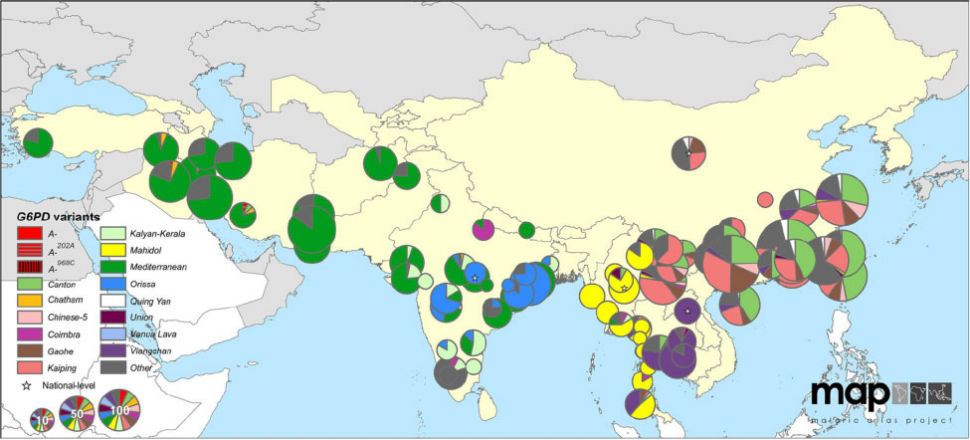
Map illustrates the diversity of common variants of G6PD deficiency in eastern Asia and dominance of Mediterranean variant in western Asia. Reproduced from Ref. 60 published under Creative Commons license.

**Figure 4.**
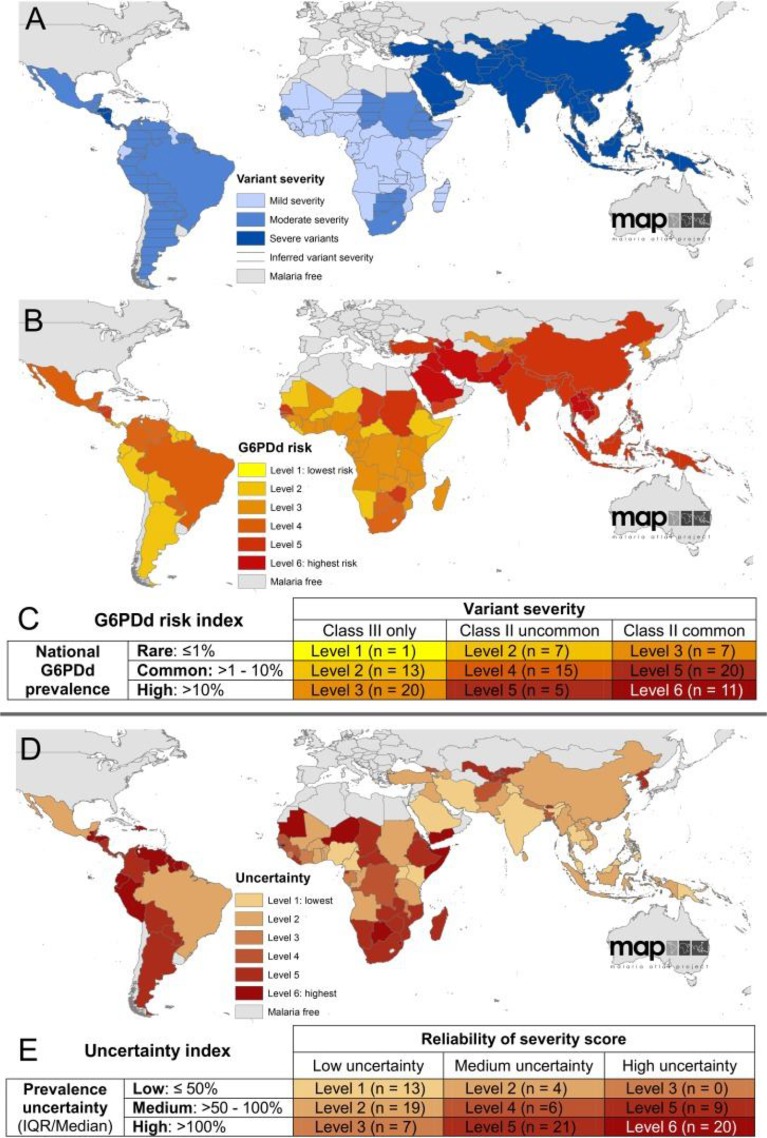
Maps illustrate geographic distribution of crude estimates of risk related to primaquine dosing derived by considering severity of impaired G6PD activity phenotype. Reproduced from Ref. 14 published under Creative Commons license.

Epidemiologists and geneticists eventually surveyed the diversity of G6PD deficiency, finding Africa and the Americas to be relatively homogeneous; the A −  variant dominated among the G6PD-deficient people on those continents. In contrast, the Mediterranean variant dominated across southern Europe, the Middle East, and through Iran, Afghanistan, Pakistan and much of western India. In eastern India, a great diversity of variants appears and extends across the rest of tropical Southeast Asia (Fig. 3).^15^,^60^,^61^ The majority of these Asian variants more closely resemble Mediterranean variant with regard to residual enzyme activity being very low (Fig. 4).^15^ The reality of serious hazard with offering primaquine without knowing G6PD status, especially in Asia, thus became recognised and acknowledged by the early 1980s.^62^ The WHO malaria treatment guidelines of 2010 expressed, ‘*In patients with the African variant of G6PD deficiency, the standard course of primaquine therapy produces a benign and self-limiting anemia. In the Mediterranean and Asian variants, hemolysis may be much more severe*’.^63^

## Therapy without G6PD Screening

Advocates of primaquine therapy without G6PD screening point to the lack of evidence demonstrating harm being done. An exhaustive review aimed at demonstrating safety of a single gametocytocidal dose of primaquine uncovered little evidence of risk of death with any primaquine therapy and estimated such at 1 in 621 428 treatments.^64^ As noted in that report, however, reliance upon evidence passively gathered through conspicuously poor pharmacovigilance systems should be cautiously considered. The hypothesis of relative safety in primaquine therapy without G6PD screening requires direct evidence that does not yet exist. Instead, the available evidence (described above) demonstrates the capacity for serious harm with therapeutic dosing of G6PD-deficient patients.

**Figure 5.**
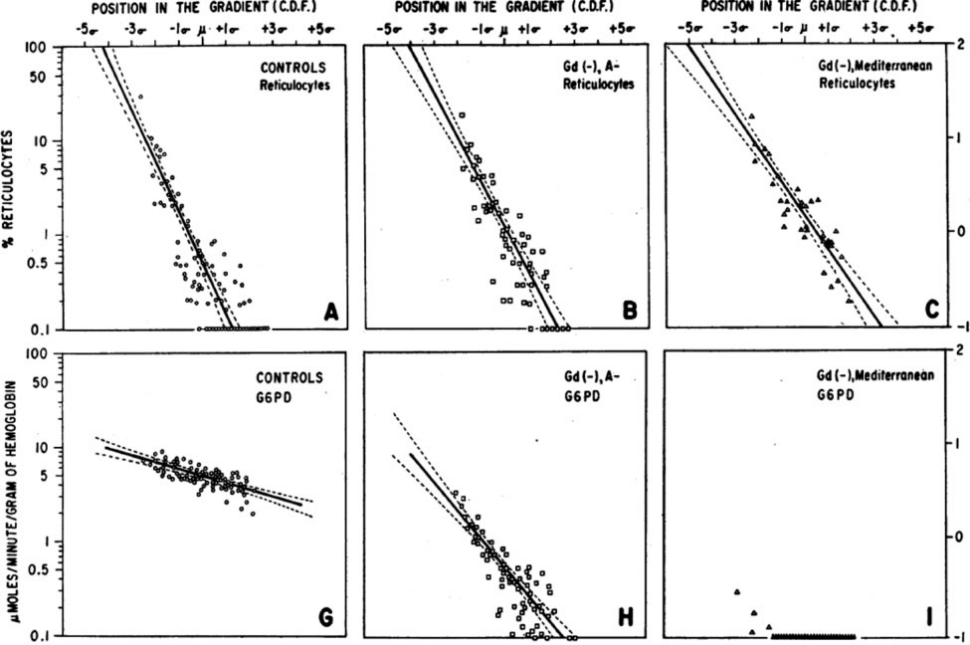
Data illustrating key variance in the extent to which G6PD activity may be impaired among variants. The *x*-axis, ‘Position in the Gradient (C.D.F.)’ represents fractions of red blood cells collected by ultracentrifugation, where the youngest cells are to the left, and oldest cells to the right. The top three panels illustrate the proportion of red blood cells as reticulocytes among G6PD-normal (panel A) and two subjects having either A −  (panel B) or Mediterranean (panel C) variants of G6PD deficiency. Panels G, H and I, illustrate relative activities of G6PD enzyme in the same age-dependent gradient. Note the conspicuous distinction between A −  and Mediterranean variants, where in the latter even reticulocytes suffer severely impaired enzyme activity. In contrast, the youngest A −  red cells have nearly normal activity. This difference largely explains the ability of A −  patients to develop tolerance to large doses of primaquine as in Fig. 6, whereas Mediterranean patients do not. Reproduced from Ref. 64 with the permission of the Journal of Clinical Investigation.

**Figure 6.**
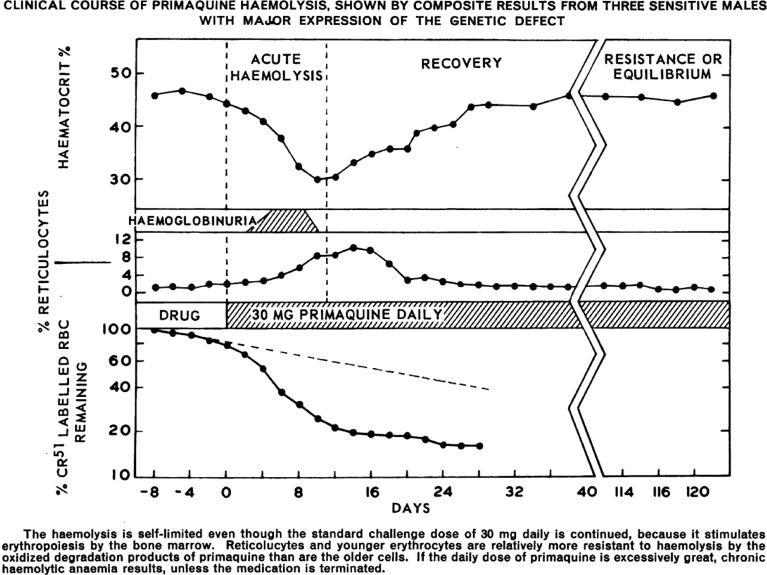
Graph illustrates development of tolerance to primaquine in three otherwise healthy African–American subjects with A −  variant of G6PD deficiency. After an initial haemolytic anaemia, reticulocytemia followed and in turn haematocrit returned to normal. The subjects then maintained effective normal blood profiles despite receiving daily doses of 30 mg primaquine for 120 days. Reproduced from Ref. 65 with permission of the Bulletin of the World Health Organization.

The diversity of G6PD deficiency is key to analysing evidence of primaquine safety. In the discovery of G6PD deficiency and its diversity, two broad phenotypes emerged: mildly primaquine sensitive, and exquisitely sensitive, those being represented by the African A −  and Mediterranean variants, respectively. Figure 5 best represents the stark distinction between them regarding residual levels of G6PD activity in red blood cell populations.^65^ The A −  red blood cells begin life with only slightly diminished G6PD activity that then more sharply declines as they age, both relative to G6PD-normal red blood cells. This largely explains the phenomenon of acquired tolerance to daily primaquine dosing in A −  variant, as illustrated in Fig. 6.^66^ Primaquine destroys older red blood cells in the first few days of dosing, and these are replaced by young red blood cells capable of surviving primaquine challenge. As may also be seen in Fig. 5, however, Mediterranean variant offers no red blood cell subpopulations capable of surviving primaquine challenge. It is known that even reticulocytes of this variant are susceptible to destruction by primaquine. Acquired tolerance of the drug in A − fashion is thus impossible among Mediterranean-like variants, and each new primaquine dose deepens the haemolytic crisis.^56^,^58^,^67^

**Figure 7.**
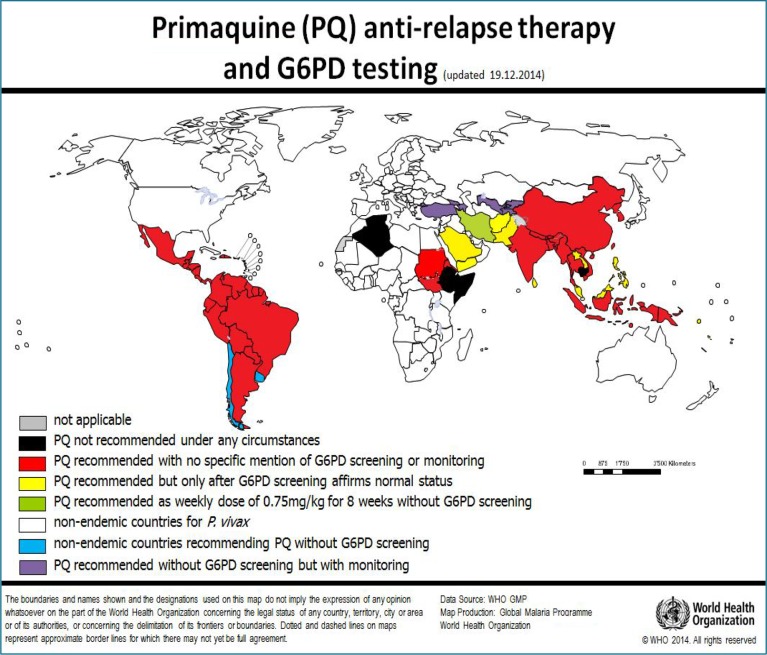
Primaquine treatment policies against relapse among endemic nations in 2014. Reproduced with permission of the World Health Organization, Global Malaria Program, Geneva.

Patients having Mediterranean-like G6PD deficiency would not likely survive a full 14-day regimen of primaquine, and death is a confirmed outcome in some patients.^68^–^70^ Onset of conspicuously dark urine after three or four daily doses of primaquine would serve to alert patients and their providers of serious trouble and to then cease dosing. This obvious sign, and understanding its relationship to the drug, has surely saved many patients from poor outcomes. However, malaria patients in the rural endemic tropics may not grasp that their deterioration with progressive haemolytic crisis (weakness, shortness of breath and jaundice) is due to primaquine rather than the recent illness diagnosed as malaria. They may steadfastly continue dosing with the drug provided to them to combat that malaria, mistaken in the belief that malaria still sickens them. They also may not notice the darkening of urine. The toilets and latrines of the rural tropics offer every opportunity to miss onset of dark urine – unlit pit outhouses represent the rule and clear water-filled toilet bowls in brightly lit rooms the exception. Offering anti-relapse primaquine therapy without knowledge of G6PD status should be acknowledged as an intrinsically dangerous practice to be undertaken only under close clinical supervision. The dominant policies guiding primaquine therapy, however, seem rooted in the ‘mild and self-limiting’ dogma of the 1960s (Fig. 7).

## The Threat of Relapse

While primaquine therapy for unscreened patients may be dangerous, so too is withholding that treatment. There is no alternative hypnozoitocide, only a stark choice between risk of haemolysis or recurrent clinical attacks. In a weighing of risk and benefit that includes a harmless clinical attack or two, few would opt for risking primaquine therapy. However, today we understand that repeated attacks of vivax malaria pose a serious threat to the health and life of patients. This reality greatly deepens the primaquine/G6PD deficiency-relapse dilemma, and examination of the threat posed by hypnozoites informs weighing of risk and benefit.

**Figure 8.**
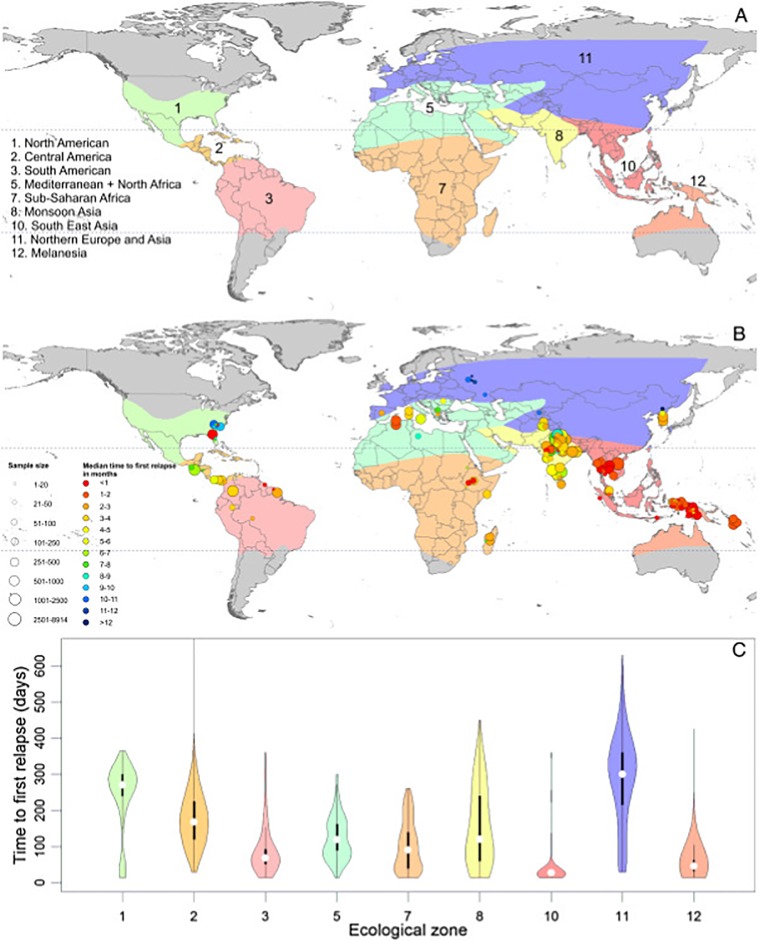
Map charts geographic variation in relapse behaviours by *Plasmodium vivax* among ecological zones and the graph below illustrates the timing and frequency of relapse within each ecological zone. Reproduced from Ref. 70 published under Creative Commons license.

The risk of relapse *per se* assigns weight of probability and frequency of subsequent clinical attack, each in turn bearing upon risk of poor clinical outcome and onwards transmission. Acknowledging the great diversity of relapse behaviours among *P. vivax* strains across regions^71^ (Fig. 8), the focus here is upon the worst-case scenario of Chesson-like *P. vivax* that occurs in Southeast Asia and the Southwest Pacific. These parasites very quickly cause relapse in almost all patients, and do so multiple times at about 2-month intervals. In contrast, among strains from India or Korea, for example, relapse occurs in a minority of patients and typically does so more than 6 months after the primary attack.

The experimental challenge work on the Chesson strain of *P. vivax* during the 1940s and 1950s left an abundant literature on its relapse behaviours.^72^ The findings effectively mirrored those in American soldiers naturally infected by *P. vivax* in the Pacific War.^73^ Relapse occurred as soon as 17 days after onset of patency of the primary attack, and by the 28th day more than one half of patients relapsed. After 8 weeks almost all had relapsed. Most patients or subjects then relapsed a second or third time, and many went on to experience 10 or more relapses.

Contemporary studies affirm that older literature regarding relapse behaviour by Chesson-like strains of *P. vivax*. The majority of patients infected in Thailand typically relapsed within 28 days of patency,^74^,^75^ as was true among a cohort of Indonesian soldiers infected in Indonesian New Guinea (Papua).^76^ The incidence density of attack by first relapse in those studies was approximately 5/person-year in the first 2 months following patency. Those patient subjects were not permitted to relapse multiple times, but a New Zealand woman infected in Papua New Guinea in 2012 suffered five relapses within a year at 2-month intervals as a consequence of therapeutic failure of primaquine (very likely caused by a cytochrome P-450 2D6 polymorphism).^77^ Southeast Asia and the Southwest Pacific harbour *P. vivax* carrying very high risk of multiple clinical attacks. In those regions residents mostly carry variants of G6PD deficiency considered ‘severe’, that is, relatively very low residual G6PD enzyme activity (Fig. 4).

The co-occurrence of relatively aggressive relapse behaviours and perhaps more virulent *P. vivax* with relatively severe G6PD variants may not be simply an unhappy coincidence. Two well known facts regarding this parasite and host enzyme may be mechanistically linked – (1) *P. vivax* invades only reticulocytes; and (2) G6PD enzyme activity naturally declines as red blood cells age, with highest levels occurring in reticulocytes (as in Fig. 5). There may be host survival advantage in forcing malaria parasites to live in a red blood cell cytosol deprived of the detoxifying effects of G6PD activity (the sole source of electrons for NADP+ and oxidised glutathione).^78^ Achieving this with *P. vivax* infection would require reticulocytes having greatly reduced G6PD activity (as in panel I of Fig. 5). If so, the unhappy coincidence is the extreme vulnerability of such red blood cell populations to destruction by primaquine – the patients most likely to gain the potentially life-saving benefits of primaquine therapy against relapse may also be those most likely to suffer serious harm by its use.

Primaquine therapy for such patients – infected by the strains of parasites most likely to cause rapid multiple relapses of deepening consequences, and harbouring the most severely deficient G6PD variants – without knowing G6PD status may be the most extreme expression of this clinical and public health dilemma. Risk of serious harm haunts either decision. Solving the dilemma, however, is relatively simple; screen out the G6PD-deficient minority and freely treat the G6PD-normal majority. Primaquine is a remarkably safe and well-tolerated drug among non-pregnant G6PD-normal patients^79^ and appears to retain superb efficacy against relapse even against Chesson-like strains.^76^

## Solving the Primaquine-G6PD Deficiency Dilemma

The gold standard for screening patients for G6PD deficiency has been the fluorescent spot test since the mid-1960s.^80^ Although relatively simple and inexpensive by the standards of most modern clinical laboratories, the test suffers several drawbacks precluding routine use where the vast majority of malaria patients obtain treatment. The kit requires a cold chain for reagents, specialised equipment (incubator, pipettor and source of ultra-violet light) and laboratory skills. At about USD4/test, it is prohibitively expensive in the rural tropics. The many complex factors explained in this review account for the lack of impetus to solve this problem with a screening kit suited to the impoverished rural tropics.

Over the decades, no research-funding agency put point-of-care G6PD diagnostics on its agenda. The problem would eventually be addressed by entrepreneurial initiative and capital. In the past 5 years, two companies developed point-of-care G6PD diagnostic devices; CareStart^®^ G6PD from AccessBio™ (Somerset, NJ, USA);^81^,^82^ and BinaxNOW^®^ G6PD from Alere™ (Waltham, MA, USA).^83^,^84^ The latter kit suffers the drawbacks of strict temperature limitations (testing above 25°C may be invalid) and relatively high cost (about $15/test). The kit from AccessBio^®^ performs well at ambient temperatures and costs only $1.50/test. Each is relatively simple to perform and delivers a result in about 10 minutes.

Rolling out any G6PD diagnostic device for routine use at the periphery of healthcare in the endemic rural tropics may finally unleash the enormous therapeutic and public health benefits of primaquine without causing harm. That task is by no means trivial, both in terms of finance/logistics and training/quality assurance. Nonetheless, there may be few other means of greatly accelerating the control and elimination of *P. vivax*. Moreover, doing so lays the foundations for safe introduction of the single-dose 8-aminoquinoline therapy called tafenoquine, a product now approaching regulatory registration and clinical availability.^85^ That product will also require excluding G6PD-deficient patients in order to realise its enormous promise as a new tool against vivax malaria.

Evidence demonstrates that the failure to prevent relapse incurs risk of serious illness and death with vivax malaria, as evidence demonstrates that treating the unscreened incurs risk of serious harm caused by primaquine. Robust G6PD diagnostic devices at the point of care would largely solve this dilemma and greatly mitigate risk of fatal outcomes provoked by the parasite or its treatment. Humanity should muster the resources and energies needed to implement those devices as an essential element of routine care for malaria. Chemotherapeutic or chemo-preventive strategies for patients who cannot receive primaquine (G6PD-deficient, pregnant women and infants) should also be conceived, optimised and validated.

## Critical Reflection

Malariology virtually abandoned research on *P. vivax* over 60 years ago, apparently confident in the conviction of harmlessness relative to *P. falciparum*, and content with the chloroquine–primaquine chemotherapeutic solution for it. No evidence emerged demonstrating *P. vivax* as dangerous and evidence seeking to prove the hypothesis of harmlessness was not pursued. This also occurred with the G6PD deficiency-primaquine problem – the absence of evidence of harm after already widespread use of the drug overruled laboratory and clinical studies pointing to the potential for serious harm with Mediterranean-like variants of G6PD deficiency.

Today, evidence and acknowledgement of these problems, along with new technical solutions, now offer the promise of correction of course. Patients suffering acute *P. vivax*, especially repeated bouts due to untreated hypnozoites, are today acknowledged to be at risk of severe and fatal illness with inadequate treatment. This understanding alone substantially improves the likelihood that such outcomes will become rare as a result of spurring the implementation of much more aggressive and effective control, prevention, diagnosis and treatment practices. That includes impelling the achievement of vastly improved access to safe primaquine therapy by point-of-care G6PD devices at the periphery of care.

The primary lesson in these events and the thinking contemporary to them demonstrates the peril in not developing and following materialised evidence. This is the core process of the scientific method, and in not applying it we risk actions based upon fallacy. In the instance of primaquine and G6PD deficiency in therapy of *P. vivax* malaria, we indeed seemed inclined to the confusion alluded to in Taleb's quote at the opening of this paper – the absence of evidence construed as evidence of absence guided strategy and practice. The assumptions embedded within those practices proved wrong and indeed led to misunderstanding and error. Over the decades, consequences have very likely accumulated – unnoticed and unexamined morbidity and mortality caused by the parasite or its treatment with primaquine. Those may finally cease with assurance of safe delivery of primaquine to patients in need of it spurred by the materialised evidence informing the necessity and urgency of doing so.

## Disclaimer Statements

**Contributors** All named authors contributed to this article.

**Funding** Wellcome Trust

**Conflicts of interest** The author is a consultant to the Medicines for Malaria Venture in the development of tafenoquine (GSK, UK) for anti-relapse therapy against *P. vivax*, and consults the WHO on issues pertaining to *P. vivax* elimination and control, in addition to containment of artimisinin-resistant *P. falciparum*.

**Ethics approval** There is no ethical approval for this article.
